# Mechanism of Oxytocin-Induced Contraction in Rat Gastric Circular Smooth Muscle

**DOI:** 10.3390/ijms24010441

**Published:** 2022-12-27

**Authors:** Mohammad Alqudah, Rima Abdul Razzaq, Mahmoud A. Alfaqih, Othman Al-Shboul, Ahmed Al-Dwairi, Safa Taha

**Affiliations:** 1 Department of Physiology, College of Medicine and Medical Sciences, Arabian Gulf University, Manama 329, Bahrain; 2 Department of Physiology and Biochemistry, College of Medicine, Jordan University of Science and Technology, Irbid 22110, Jordan; 3 Department of Biochemistry, College of Medicine and Medical Sciences, Arabian Gulf University, Manama 329, Bahrain; 4 Department of Molecular Medicine, Princess Al-Jawhara Centre for Molecular Medicine, College of Medicine and Medical Sciences, Arabian Gulf University, Manama 329, Bahrain

**Keywords:** Oxytocin, smooth muscle contraction, atosiban

## Abstract

Oxytocin produces an excitatory effect on gastric muscle through the activation of receptors present on stomach smooth muscle cells. However, the intracellular mechanisms that mediate oxytocin excitatory effects are still largely unknown. Therefore, we aimed to investigate the signaling pathways involved in oxytocin-induced contractions in gastric smooth muscle, shedding light on phospholipase C (PLC)-β1 signaling and its downstream molecules, including inositol 1,4,5- trisphosphate (IP_3_) and myosin light chain kinase (MLCK). The contractions of gastric smooth muscle from male rats were measured in an organ bath set up in response to exogenous oxytocin 10^−7^ M, in the presence and absence of inhibitors of the indicated signaling molecules. Oxytocin (10^−9^–10^−5^ M) induced dose-dependent stomach smooth muscle contraction. Pre-incubation with atosiban, an oxytocin receptor inhibitor, abolished the oxytocin-induced contraction. Moreover, PLC β1 inhibitor (U73122) and IP_3_ inhibitor Xestospongin C inhibited oxytocin-induced muscle contraction to various degrees. Verapamil, a calcium channel blocker, inhibited oxytocin-induced contraction, and pre-incubation of the strips, with both verapamil and Xestospongin C, further inhibited the excitatory effect of oxytocin. Chelation of intracellular calcium with BAPT-AM (1,2-bis-(o-aminophenoxy) ethane-N,N,N′,N′-tetraacetic acid) significantly inhibited the effect of oxytocin on muscle contraction. Finally, pre-incubation of the strips with the Ca^2+^/calmodulin-dependent protein kinase selective inhibitor STO-609 significantly inhibited the contraction induced by oxytocin. These results suggest that oxytocin directly stimulates its cell surface receptor to activate PLC β1, which in turn liberates IP_3_, which eventually elevates intracellular calcium, the prerequisite for smooth muscle contraction.

## 1. Introduction

Oxytocin is a hormone that is associated with parturition and lactation [1]. Oxytocin performs its biological functions by activating its cell surface receptor, the oxytocin receptor (OXR) [2]. OXR is a G protein-coupled receptor that is coupled to Gαq protein. The binding of oxytocin to its receptor leads to activation of phospholipase C β and the subsequent liberation of diacylglycerol (DAG) and phosphatidylinositol triphosphate (IP_3_) [3]. Both molecules are essential for intracellular Ca^2+^, which is the final signal for oxytocin on effector cells [4]. Elevation of intracellular Ca^2+^, activation of calcium calmodulin kinase, and the subsequent activation of smooth muscle contraction machinery suggests that oxytocin can interact with the activation of smooth muscle contraction in settings other than its conventional sites of action. One of the sites where oxytocin has been implicated to contribute to its function is the gastrointestinal tract [5]. There is a wealth of data investigating the role of oxytocin in food intake, glucose homeostasis, and the gut–brain axis. These functions are mostly caused by the central actions of oxytocin in different brain regions [6]. In this report, we are concerned with the exogenous actions of oxytocin in the gastrointestinal tract (GIT).

Oxytocin and its receptor are expressed in different areas of the GIT of humans, rats, rabbits, and mice [7,8,9,10]. Oxytocin receptors have been identified in GIT enteric neurons, epithelial cells, and smooth muscle cells, suggesting that oxytocin could interfere with multiple functions of the GIT [8]. Interestingly, OXR is expressed on both longitudinal and circular smooth muscle cells of rat stomachs, thus suggesting there is oxytocin involvement in fine-tuning GIT motility [8]. The functions of oxytocin in GIT motility are not clear. Some studies have indicated an acceleratory effect, while others report an inhibitory effect. Oxytocin is released in healthy individuals after a fatty meal, which leads to increased gastric emptying time and satiety [11]. This effect has been investigated and was found to be the result of cross-talk between oxytocin and cholecystokinin. Moreover, oxytocin leads to the restoration of gastric motility in mice following chronic homotypic stress through the central inhibition of the hypothalamic corticotropin-releasing factor [12]. However, other studies have shown that oxytocin does not affect gastric emptying and fluid intake in healthy subjects after a liquid meal [13]. Oxytocin showed inhibitory effects on colonic contractions in rats, and that effect was enhanced by stress stimuli, such as cold temperatures [5]. The same study showed that the inhibitory effect of oxytocin was realised through its action on enteric neurons and estrogen-dependent conditions [5].

Exogenous oxytocin treatment affects gastric pressure, with an early transient decrease and late increase in gastric pressure. This dual effect can be explained by the effect of oxytocin on cholecystokinin release, which leads to reduction in gastric pressure, while the late effect is mostly due to the direct effect of oxytocin on gastric smooth muscle contractile function [8]. Moreover, oxytocin stimulates the peristaltic reflex in healthy women’s activated colon [14]. In contrast, the inhibition of colonic activity was demonstrated via in vivo treatment of proximal colonic rabbit muscle strips [10], and a similar effect was observed in the colonic motor activity of stressed rats [15]. These contradicting results might reflect species-related differences, regional differences, and central versus peripheral oxytocin sources. However, the effect of oxytocin on GIT motility deserves further investigation to elucidate the exact mechanism of action, especially the direct effect of GIT smooth muscle cells.

Although several studies have investigated the excitatory role of oxytocin in GIT, its effect on GIT smooth muscle PLC-β1 and intracellular calcium elevation and subsequent contractions have not been examined. Therefore, we hypothesize that oxytocin acts directly on gastric smooth muscle cells to stimulate its receptors, which in turn activates PLC-β1, which eventually leads to the activation of Ca^2+^/calmodulin-dependent myosin light chain kinase (MLCK), which then phosphorylates MLC20, leading to gastric muscle contraction.

## 2. Results

**Figure 1:**
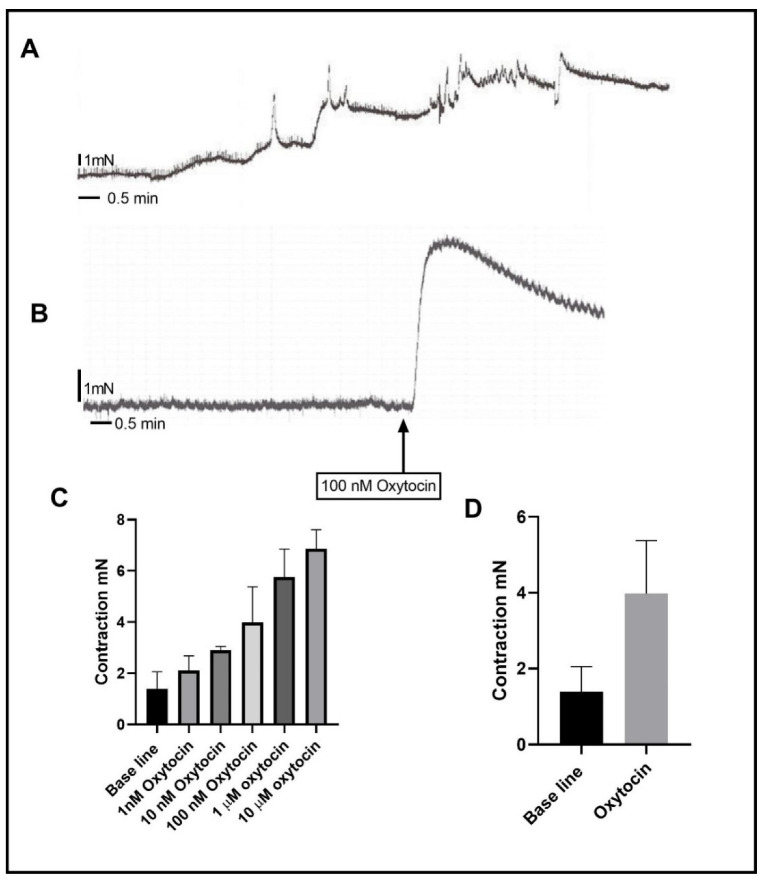
Effect of oxytocin on rat circular muscle strips from the gastric antrum. (**A**) is a summary of the effect of oxytocin on gastric smooth muscle strip. Oxytocin dose-dependently produced contraction in the range of 10^−9^ to 10^−5^ M. (**B**) is a representative trace illustrating that 100 nM oxytocin induced contraction in gastric circular muscle strips. (**C**) is a summary graph showing that oxytocin treatment dose-dependently significantly increased the contraction. (**D**) is a summary graph trace illustrating that 100 nM oxytocin induced contraction in gastric circular muscle strips. Data expression is in mN of force. Values are mean ± SEM. *n* = 11.

In rat stomach antrum circular muscle strips, oxytocin, in the range of 1 nM to 10 µM, caused a concentration-dependent contraction (Figure 1). The maximum response (6.864 ± 0.606 mN) was achieved at 10 µM of oxytocin. The effect of all inhibitors was tested by using a submaximal dose of oxytocin at 100 nM (Figure 1), which induced a significantly larger contraction (3.977 ± 0.466 mN,) in comparison to the mean baseline tension (1.396 ± 0.466 mN) (*n* = 11, *p* < 0.0001). The contraction induced after pretreatment with the inhibitors was compared with that of 100 nM of oxytocin (control).

### 2.1. Effect of Oxytocin Receptor (OR) Inhibition on Oxytocin-Induced Contraction

**Figure 2:**
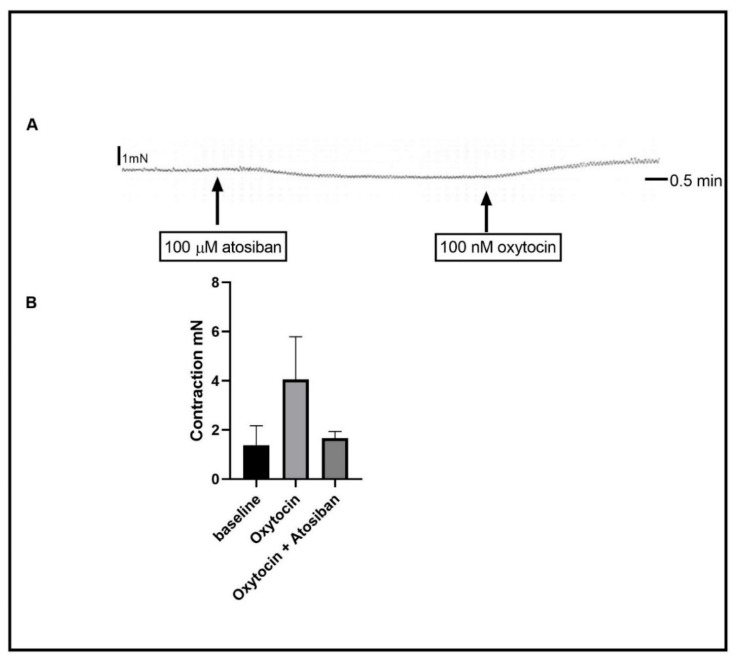
Effect of oxytocin receptor (OR) inhibition on oxytocin-induced contraction. (**A**) is a representative trace demonstrating 100 nM oxytocin-induced contraction after 15 min of incubation with 100 µM of atosiban. It also shows that atosiban did not affect the baseline activity. (**B**) is a summary graph showing oxytocin-induced contraction after incubation with atosiban. Data expression is in mN of force. Values are mean ± SEM. *n* ≥ 11.

Oxytocin mediates its physiological effect by activating its cell surface receptor. To test the role of OR, atosiban, a selective OR inhibitor, was used. Atosiban (100 µM) was added 15 min before oxytocin was added to the organ bath. Atosiban abolished the contraction induced by oxytocin (Figure 2) (Basal activity (*n* = 11) vs. oxytocin (13) vs. oxytocin + atosiban (5), 1.377 ± 0.790 mN vs. 4.052 ± 1.700 mN vs. 1.672 ± 0.270 mN, and *p* < 0.0001). Similarly, the incubation of the strips with the selective OR antibodies (ab300443, 1:100) prevented the excitatory effect of oxytocin on the muscle strips (Figure S1). During the 15 min incubation with atosiban, the basal tone was not affected. In pre-incubation of the gastric antrum strips with either the muscarinic ACh blocker, atropine, or with the sodium channel blocker, tetrodotoxin did not affect the oxytocin-induced contraction (data not shown).

### 2.2. Effect of Phospholipase C (PLC) Inhibition

**Figure 3:**
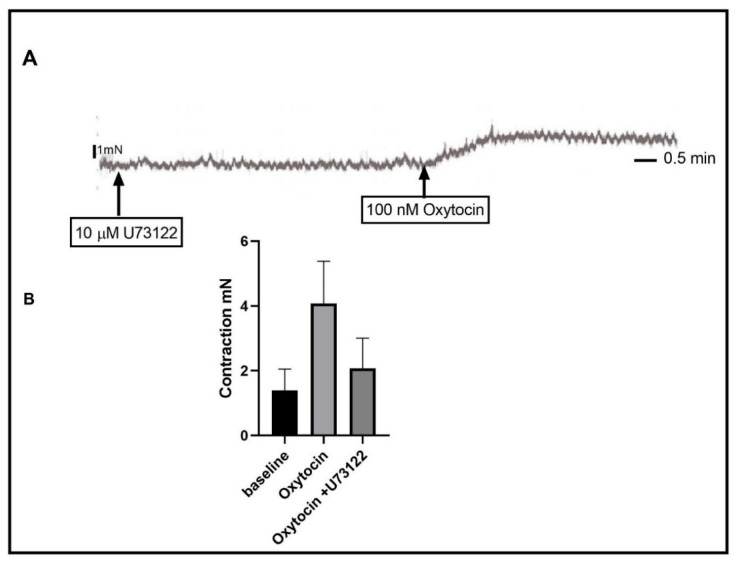
Effect of phospholipase C (PLC) inhibition on oxytocin-induced contraction. (**A**) is a representative trace demonstrating 100 nM oxytocin-induced contraction after 15 min of incubation with 10 µM of U73122. It also shows that U73122 did not affect the baseline activity. (**B**) is a graph summarizing the oxytocin-induced contraction after incubation with U73122. Data are expressed in mN of force. Values are mean ± SEM. *n* ≥ 35.

The activation of the OR receptor is coupled to Gq/G11, which leads to the activation of PLC and the generation of second messengers. To test the effect of PLC inhibition, we pretreated the organ bath with U73122, an agent that blocks the activity of PLC. The addition of U73122 15 min before treatment with oxytocin resulted in significant inhibition of oxytocin-induced contractions. See Figure 3 (basal activity vs. oxytocin vs. oxytocin+ U73122, 1.396 ± 0.418 mN vs. 4.081 ± 0.434 mN vs. 2.078 ± 0.418 mN, *n*_(total)_ = 35, *p*-value < 0.0001).

### 2.3. Effect of Blocking Different Sources of Calcium

**Figure 4:**
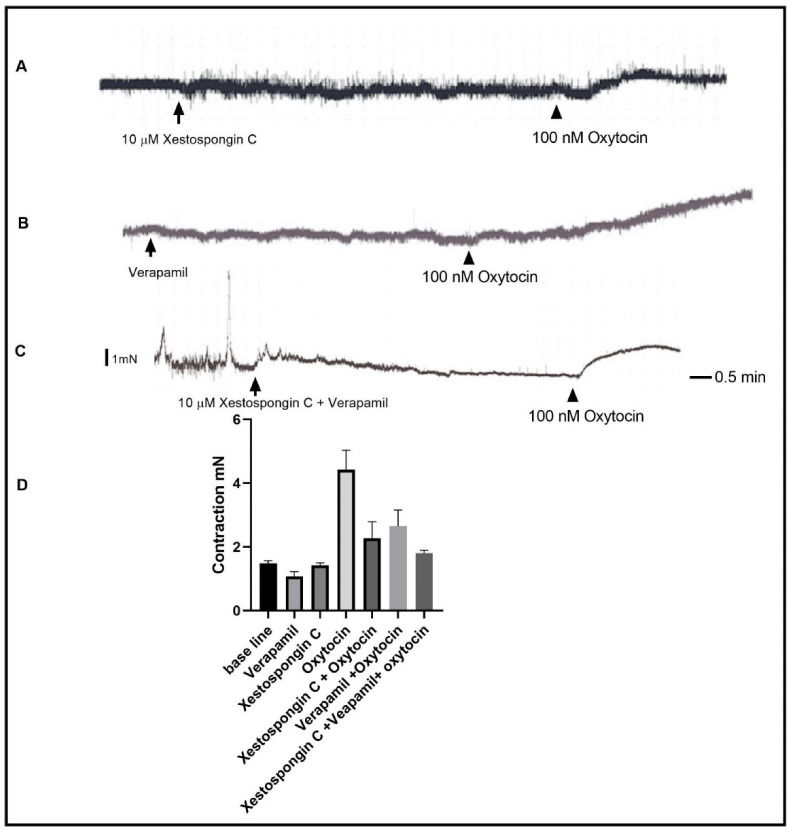
Effect of blocking IP_3_ receptor and VGCC on oxytocin-induced contraction. (**A**) is a representative trace demonstrating 100 nM oxytocin self-induced contraction after 15 min of incubation with 10 µM of Xestospongin C. It also shows that Xestospongin C did not affect the baseline activity. (**B**) is a representative trace demonstrating 100 nM oxytocin-induced contraction after 15 min of incubation with 10 µM of verapamil. It also shows the verapamil effect on the baseline activity. (**C**) is a representative trace demonstrating 100 nM oxytocin-induced contraction after 15 min of incubation with both 10 µM of Xestospongin C and 10 µM of Verapamil. It also shows the effect on basal activity. (**D**) is a summary graph showing that oxytocin-induced contraction is significantly inhibited by Xestospongin C and by Verapamil and that the inhibition was greater when both agents were used. Data expression is in mN of force. Values are mean ± SEM. *n* = 7.

**Figure 5:**
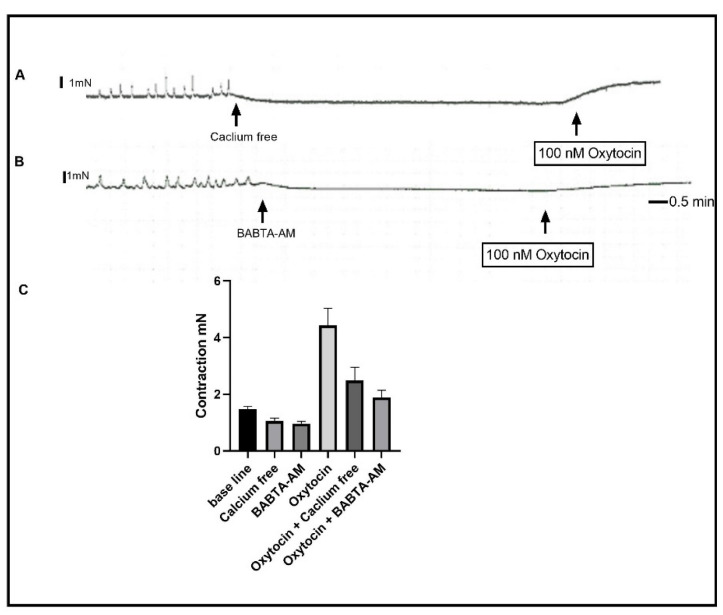
Effect of calcium-free Krebs buffer and chelating cytosolic calcium on oxytocin-induced contraction. (**A**) is a representative trace illustrating oxytocin-induced contraction in calcium-free Krebs buffer. (**B**) is a representative trace illustrating oxytocin-induced contraction in the presence of 10 µM of BABTA-AM. (**C**) is a summary graph showing that oxytocin-induced contraction is significantly inhibited by removing extracellular and intracellular calcium. Data expression is in mN of force. Values are mean ± SEM. *n* = 6.

IP_3_ receptor activation and the elevation of cytosolic calcium are, together, a downstream signaling of PLC activation. We tested the role of the IP_3_ receptor by using the IP_3_ selective inhibitor Xestospongin C. Pre-incubation of the muscle strips with 10 µM of Xestospongin C inhibited oxytocin-induced contraction to 66% less than the control value (Figure 4) (Basal activity vs. Xestospongin C vs. oxytocin vs. oxytocin + Xestospongin C: 1.484 mN vs. 1.077 mN vs. 4.425 mN, vs. 2.275 mN; SEM of difference is 0.279, *n*_(total)_ = 16, *p* < 0.0001). The remaining contraction suggested that oxytocin induced the elevation of cytosolic calcium via other means. Thus, we tested the role of calcium influx by inhibiting the influx of calcium through voltage-gated calcium channels using the selective inhibitor verapamil. A total of 10 µM of verapamil in the organ bath 15 min before oxytocin addition resulted in the inhibition of oxytocin-induced contractions by 40%. (Figure 4, Basal vs. verapamil vs. oxytocin vs. oxytocin+ verapamil: 1.484 mN vs. 1.807 mN vs. 4.425 mN vs. 2.650 mN, SEM of difference is 0.279, *n*_(total)_ = 16, *p* = <0.0001). Preincubation of the muscle strips with both Xestospongin C and verapamil inhibited the basal tone contraction level and abolished the phasic basal activity. Moreover, the pre-incubation of the muscle strips with both Xestospongin C and Verapamil resulted in further inhibition of contraction at a greater level (Figure 4, Xestospongin C and Verapamil contraction 1.807 ± 0.292 mN). The addition of oxytocin to the organ bath in a calcium-free Krebs buffer resulted in a contraction similar to that obtained with the presence of verapamil (Figure 5) 2.475 ± 0.235 mN, *n* = 4, *p* < 0.0001. Finally, pre-incubation of the muscle strips with 10 µM of the membrane-permeable calcium chelating agent BABTA-AM resulted in contraction inhibition similar to that seen in the presence of both Xestospongin C and verapamil (Figure 5) 1.875 ± 0.235 mN, *n* = 4, *p* < 0.0001.

### 2.4. Effect of Ca^2+^/Calmodulin-Dependent Protein Kinase Kinase (CaM-KK) Inhibition

**Figure 6:**
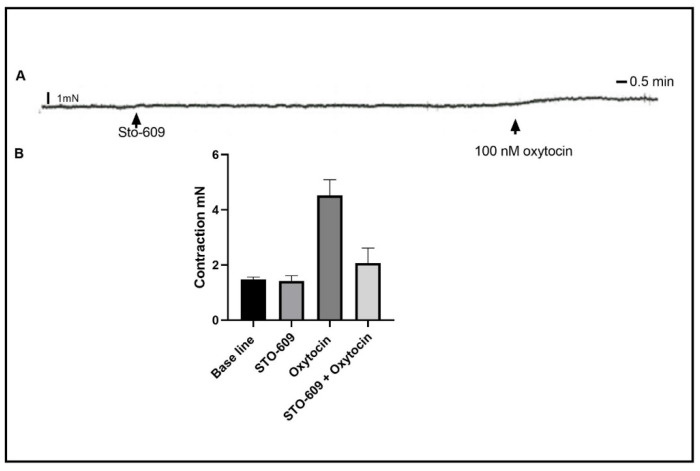
Effect of inhibition of Ca^2+^/calmodulin-dependent protein kinase (CaM-KK) on oxytocin-induced contraction. (**A**) is a representative trace illustrating oxytocin-induced contraction after 30 min of pre-incubation with the CaM-KK inhibitor STO-609. (**B**) is a summary graph illustrating oxytocin-induced contraction significantly inhibited by STO-609. Data expression is in mN of force. Values are mean ± SEM. *n* = 4.

The elevation of cytosolic calcium concentration leads to the activation of calcium CaM-KK, which leads to MLCK activation and the subsequent activation of smooth muscle contractions. To test the role of CaM-KK in oxytocin-induced contractions in a gastric circular muscle contraction, we inhibited the CaM-KK using the selective inhibitor STO-609. Exactly 30 min of pre-incubation of the strips with 10 µM of STO-609 significantly inhibited the contraction induced by oxytocin (Figure 6), 2.067 ± 0.253 mN vs. control contraction of 4.520 ± 0.253 mN, *n*_(total)_ = 21, *p* < 0.0001.

## 3. Discussion

In this study, the functional role of oxytocin in stomach contractility was demonstrated. Oxytocin-induced antral stomach contractions are a result of activation of oxytocin receptors that are most likely located on smooth muscle cells. The effect of oxytocin is partly calcium-dependent and involves the activation of the PLC pathway.

The present excitatory effect of oxytocin on rat gastric antrum strips has been previously reported in few investigations [8,16,17]. Oxytocin increased muscle contraction in a dose-dependent manner in strips from rats’ gastric bodies, antrum, and pyloric sphincter [8]. The sodium channel blocker (tetrodotoxin) and the muscarinic ACh receptor blocker (atropine) did not affect the excitatory effect of oxytocin on gastric muscle. However, atosiban (oxytocin receptor antagonist) abolished the effect of oxytocin on muscle contraction. In accordance with these effects, we have demonstrated that atosiban and oxytocin receptor antibodies blocked the contractile effect of oxytocin on gastric antral circular smooth muscle strips. The excitatory effect of oxytocin has been reported for other regions of the gastrointestinal tract. For instance, Atef and Mona (2012) demonstrated that low doses of oxytocin produced contractions in duodenal muscle strips of female rats [18]. However, other studies reported an inhibitory effect of high doses of oxytocin on duodenal contractions [18,19,20,21]. This inhibitory effect, contradictory to findings in the present study, can be explained by the use of different experimental designs, species, GI region, or by the differential modes of action of oxytocin. In the present study, exogenous oxytocin produced an excitatory effect in mucosa-free antral muscle strips from male rats. However, in other reports, whole muscle strips were utilized, where the mucosa would affect the action of oxytocin. Moreover, some of the previous reports demonstrated that in terms of in vivo actions of oxytocin, several interactions can take place and change the role of oxytocin on GI tract motility. Cholecystokinin and androgens are among the most important hormones that interact with oxytocin effects [18,22]. Furthermore, the central actions of oxytocin and the interactions between oxytocin and the myriad of hormones and neuropeptides could result in actions on the GI tract motility other than our reported effect. Moreover, because the rationale of this study was to investigate the direct mechanism of exogenous oxytocin on stomach smooth muscle contractions, these effects and scenarios are out of the scope of this study.

The reported excitatory effect of oxytocin on gastric muscle could serve a potential therapeutic pharmacological agent to treat GIT motility disorders, especially those associated with stress, such as the delay in gastric emptying. There are many clinical symptoms of gastric emptying delay, such as discomfort, bloating, nausea, and vomiting [23,24]. In support of the beneficial effect of oxytocin to treat stress-related GIT motility disorders, Jiang and Travagli (2020) showed that oxytocin plays a fundamental role in the hypothalamic-vagal projections to control gastric motility in response to stress [23]. In a double blind, placebo-controlled pilot trial, nasal inhalation of oxytocin ameliorated constipation in women with refractory constipation [25].

Oxytocin mediates its biological functions by activating its cell membrane’s receptor, a G protein-coupled receptor that is coupled to Gq/G11 GTP binding proteins [26]. After binding to its receptor, oxytocin leads to the activation of PLC and the subsequent liberation of inositol 1,4,5-trisphosphate (IP_3_), and diacylglycerol (DAG) [27]. Here, we demonstrated that pretreatment with U73122 (a PLC inhibitor) inhibited oxytocin-induced contraction by about 40%, suggesting that there are other excitatory mechanisms of oxytocin in gastric smooth muscle. These other possible mechanisms to increase excitability and to elevate intracellular calcium by oxytocin could be similar to those found in the myometrium, whose contractile machinery components are similar to those in the GIT [28]. Such mechanisms may include the inhibition of Na^+^ channels and the activation of K^+^ channels that eventually lead to calcium influx through VGCC, a well-known process in myometrium contraction by oxytocin [29]. Moreover, MLC_20_ phosphorylation by calcium-independent MLCK [30] and the inhibition of MLC phosphatase by the Rho kinase pathway [31,32,33] are well-known contractile mechanisms in gut smooth muscle cells and are known to be activated by oxytocin in other systems [34].

We then investigated the sources of calcium for oxytocin-induced contraction. In a calcium-free buffer, oxytocin induced contraction inhibition to about 30% of the control values. A similar contraction was produced by oxytocin in strips that were pretreated with the calcium channel inhibitor verapamil. These data suggest that oxytocin-induced contraction depends on intracellular calcium stores, in addition to extracellular calcium in gastric smooth muscle. In GIT smooth muscle cells, agonist-induced contractions in the absence of extracellular calcium are very well documented [35,36]. Stimulants in this scenario increase intracellular calcium through release from internal stores via IP_3_ receptors and ryanodine receptors [36,37]. Moreover, oxytocin activates its receptor in the myometrium and in other tissues, which leads to the elevation of intracellular calcium and contraction without any calcium influx [38,39].

Inhibition of the IP_3_ receptor resulted in inhibition of the oxytocin-induced contraction in these strips, and the inhibition was similar to the well-documented role of IP_3_ receptors in acetylcholine-induced contraction in GIT smooth muscle cells [30]. The activation of the IP_3_ receptor downstream of the oxytocin receptor is very common in many systems, as well [38,40]. The activation of gastric strips in a calcium-free medium in the presence of an IP_3_ receptor inhibitor profoundly inhibited the contraction. However, it did not abolish it, suggesting that there might be other mechanisms of contraction activated by oxytocin. The activation of the muscle strips with oxytocin in the presence of both the IP_3_ receptors inhibitor and the calcium chelating agent BABTA-AM produced very weak contractions, which indicates the strong dependence of contractions on cytosolic calcium. However, the presence of residual contractions in this design suggests that oxytocin might induce completely calcium-independent contractions. The oxytocin-induced calcium-independent contraction has been described before in the smooth muscle of the uterus and other organs [41].

The inhibition of Ca^2+^/calmodulin-dependent protein kinase kinase (CaM-KK) reduced the oxytocin-induced contraction to a level similar to that seen in the presence of the calcium chelating agent, confirming the role of cytosolic calcium and the classical downstream molecules in contraction pathways, while also implying the participation of a Ca^2+^-independent MLCK.

## 4. Material and Methods

### 4.1. Preparation of Smooth Muscle Strips

Rats weighing 150–200 g were raised and housed in the animal facility administrated by Arabian Gulf University (AGU). All procedures were approved by the Institutional Animal Care and Use Committee of the AGU (No. E018-PI-6/21).

Rats were killed by injection of euthasol (100 mg/kg), and gastric muscle strips were prepared from the antral region as previously described [42]. The effect of oxytocin on stomach smooth muscle contraction was assessed using the in vitro organ bath setup [18,19]. The stomach was removed, flushed with Krebs buffer, and placed in warmed (37 °C) Krebs buffer of the following composition (in mM): 118 NaCl, 4.75 KCl, 1.19 KH_2_PO_4_, 1.2 MgSO_4_, 2.54 CaCl_2_, 25 NaHCO_3_, 11 mM glucose (pH 7.4) and bubbled with 95% O_2_-5% CO_2_. Two 3 cm-long segments in the circular direction were excised and freed of fat and mesenteric attachments. The mucosal layer was scraped with a spatula to form a circular muscle strip preparation and held in oxygenated Krebs buffer until use. Separate strips were used for each experiment and procedure. Muscle strips were tied in the orientation of the circular muscle layer at both ends with surgical silk. One end was tied to a hook, and the strip was placed in a horizontal orientation in a 5-mL organ bath (DMT -820MO) containing oxygenated and warmed Krebs buffer. The other end of the muscle strip was then attached to a force-displacement transducer. The bath fluid was changed at 15 min intervals, during equilibration and after each test agent. The force recording data were rendered using a data acquisition hardware unit (PowerLab 4/3; ADInstruments, Bella Vista, New South Wales, Australia) and LabChart 8 software (ADInstruments, Bella Vista, New South Wales, Australia).

### 4.2. Experimental Design and Data Analysis for Tension Experiments

The strips were allowed to equilibrate at 1 g tension for at least 1 h before any experiments took place. Exposure to test agents, inhibitors, and acetylcholine (ACh), high K^+^ (80 mM), and oxytocin was done while strips were suspended in the organ bath. In each case, control responses to oxytocin were first established before the subsequent addition of test agonists. Each strip served as a control for its own treatment. Experiments were conducted in the following manner. Two strips were assigned for each experimental condition and examined in parallel in separate organ baths after standard responses were obtained. Control strips were first tested with a defined concentration of ACh or high K^+^ for 3 min, then washed a total of 3 times at 15 min intervals. A second dose of ACh or high K^+^ was then applied, and the second cycle of 3 washings was undertaken. At the end of the experiment, the strips were challenged with a third dose of ACh or high K^+^ to ensure that the muscle strips retained their original activity throughout the experiment. The strips were maintained in Krebs for 1 h, and the response to ACh was repeated. Following this, a dose–response curve to oxytocin was generated in the same strips tested with ACh. The procedure was repeated with a submaximal dose of oxytocin, in the presence and absence of inhibitors of the oxytocin receptor and the PLCβ1 signaling pathway. The inhibitors were added 15–30 min before oxytocin. These inhibitors included an oxytocin receptor antagonist (atosiban (Tocris, Bristol, UK)), OXR antibody (ab300443, Abcam, Cambridge, UK), PLC β1 inhibitor (U73122, Tocris, Bristol, UK) intracellular calcium **(**1,2-Bis(2-aminophenoxy) ethane-N,N,N′,N′-tetraacetic acid tetrakis (acetoxymethyl ester (BABTA-AM), Tocris, Bristol, UK), IP_3_ inhibitor (Xestospongin C) (Tocris, Bristol, UK), and Ca^2+^/calmodulin-dependent protein kinase (CaM-KK) STO-690 (Tocris, Bristol, UK).

Strips were randomly assigned to the experimental groups. The results were analyzed using LabChart 8 software, where the basal tone was measured as the mean tension during a 3 min period, following at least 30 min of equilibration (control conditions) or 15–30 min of inhibitor incubation, before the addition of the oxytocin treatment. The basal recording was obtained during the interval just preceding oxytocin administration. Peak contraction was measured as the amplitude (peak to peak) of contraction above the basal (in grams) during the 2 min period following administration of oxytocin. The contraction amplitudes were compared between treatment conditions and controls; thus, each strip served as its own control.

### 4.3. Statistical Analysis Was Done on the Data as Recorded in Grams Force, Using ANOVA and a Post Hoc Tukey’s Test for Multiple Comparisons with GraphPad (PRISM/GraphPad Software, La Jolla, CA, USA)

A paired Student’s *t*-test was used when both control and experimental data were obtained from the same strip, and a single comparison was made. In this regard, a probability of *p* value less than 0.05 was considered significant. Values were reported in millinewton (mN) as means ± SEM. As separate strips were used from separate animals for each experiment, n values represent the number of experiments, strips, and animals.

## 5. Conclusions

In conclusion, the present study demonstrates that exogenous oxytocin activates its smooth muscle cell surface receptor, which belongs to the G-protein coupled receptor family. This leads to the activation of PLC, which in turn generates the second messenger IP_3_ that eventually leads to the elevation of intracellular calcium, the major final signal of MLC-20 phosphorylation, which represents the prerequisite for smooth muscle contraction. These data could provide new avenues to treat gastrointestinal tract disorders, especially those associated with stress and inflammatory bowel disease.

## Data Availability

The data presented in this study are available on request from the corresponding author.

## Supplementary Materials

The following supporting information can be downloaded at: https://www.mdpi.com/article/10.3390/ijms24010441/s1, Figure S1: Effect of oxytocin receptor (OR) inhibition with OR antibody on oxytocin-induced contraction.
