# Supplementary material for: Mechanism of Oxytocin-Induced Contraction in Rat Gastric Circular Smooth Muscle
Source: Int J Mol Sci. 2022 Dec 27;24(1):441. doi: 10.3390/ijms24010441 (PMC9820280; doi:10.3390/ijms24010441)
Supplement: Supplementary file 1 [file ijms-24-00441-s001.zip › ijms-2071862-supplementary.pdf]

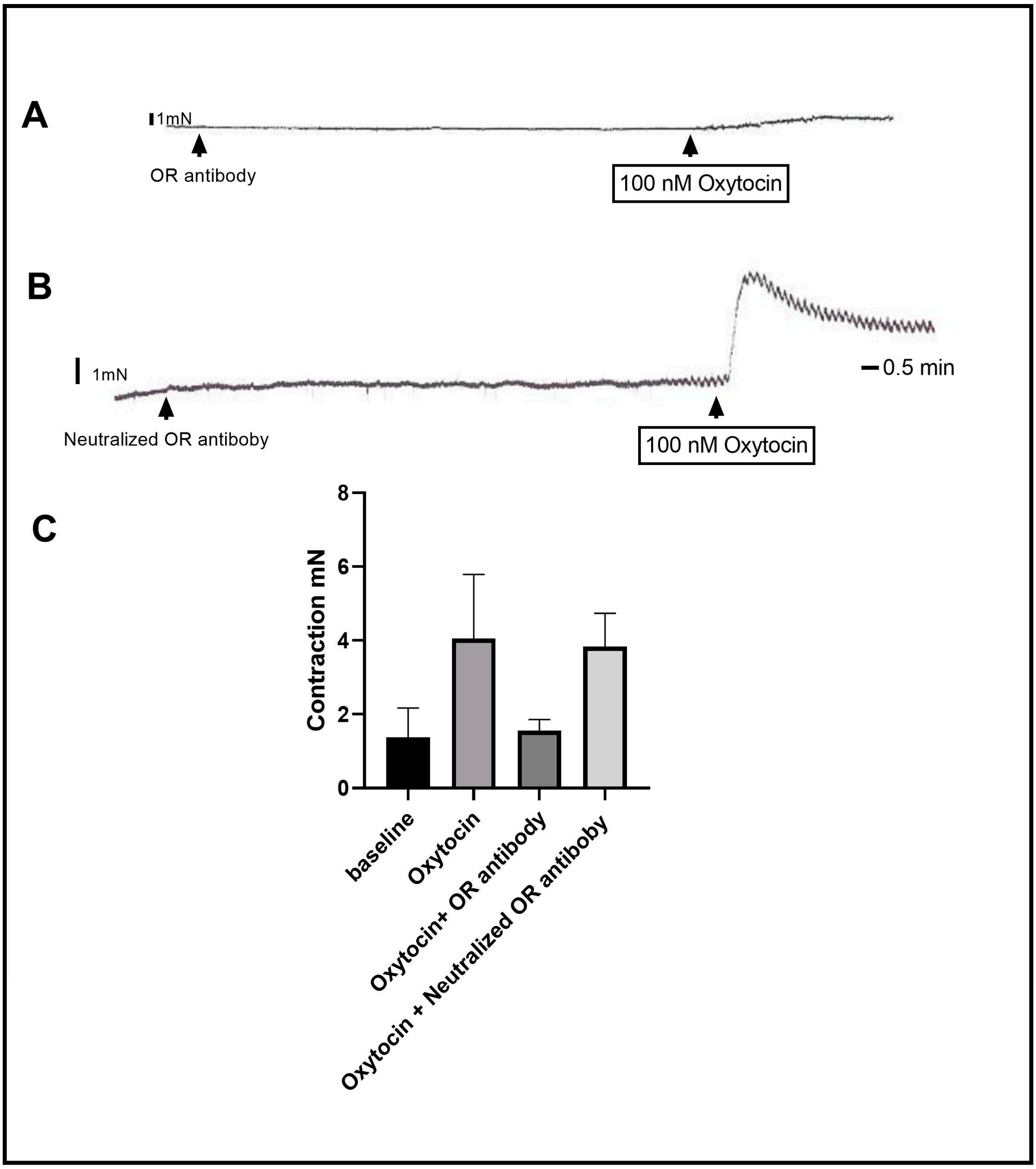

Figure S1. Effect of oxytocin receptor (OR) inhibition with OR antibody on oxytocin-induced contraction.
